# Paroxysmal atrial fibrillation recognition based on multi-scale wavelet *α*-entropy

**DOI:** 10.1186/s12938-017-0406-z

**Published:** 2017-10-23

**Authors:** Yi Xin, Yizhang Zhao

**Affiliations:** Department of Biomedical Engineering, Beijing Institute of Technology, Beijing, 100081 China

**Keywords:** PAF, HRV analysis, Multi-scale wavelet entropy, SVM

## Abstract

**Background:**

This study proposed an effective method based on the wavelet multi-scale *α*-entropy features of heart rate variability (HRV) for the recognition of paroxysmal atrial fibrillation (PAF). This new algorithm combines wavelet decomposition and non-linear analysis methods. The PAF signal, the signal distant from PAF, and the normal sinus signals can be identified and distinguished by extracting the characteristic parameters from HRV signals and analyzing their quantification indexes. The original ECG signals for QRS detection and HRV signal extraction are first processed. The features from the HRV signals are extracted as feature vectors using the wavelet multi-scale entropy. A support vector machine-based classifier is used for PAF prediction.

**Results:**

The performance of the proposed method in predicting PAF episodes is evaluated with 100 signals from the MIT-BIT PAF prediction database. With regard to the dynamics and uncertainty of PAF signals, our proposed method obtains the values of 92.18, 94.88, and 89.48% for the evaluation criteria of correct rate, sensitivity, and specificity, respectively.

**Conclusions:**

Our proposed method presents better results than the existing studies based on time domain, frequency domain, and non-linear methods. Thus, our method shows considerable potential for clinical monitoring and treatment.

## Background

Atrial fibrillation (AF) is the most common cardiac arrhythmia; it has complicated causes and categories. AF reduces the cardiac function and increases the risks of stroke and thrombus [1]. Almost half of AF patients are paroxysmal atrial fibrillation (PAF) patients. If not treated timely, PAF might transform into permanent AF. Early treatment of PAF leads to a low relapse rate; therefore, the study of PAF is both theoretically and clinically significant.

The onset and termination of PAF are coupled with autonomic dysfunction. As a valid method of non-invasive evaluation, heart rate variability (HRV) analysis is an effective approach to reflect the characteristics of the autonomic nervous system. HRV-analysis-based AF automatic identification is a popular direction in AF diagnosis [2]. The HRV signal can be a substantial factor in the field of AF classification.

Heart rate variability analysis can be divided into two kinds: parameter-based and non-parametric models. Some examples include time domain, transform-domain feature, complexity feature analysis, and non-linear feature analysis [3–5]. These methods mostly concentrate on mining the successive heart rate regulation binging using the auto rhythmicity modulation of the cardiac muscle cell from the autonomic nervous.

Some researchers currently apply multi-scale analysis methods, such as wavelet transform(WT) and empirical mode decomposition (EMD),to analyze HRV and effectually distinguish signal and noise. Noise jamming is eliminated and the HRV signal mining algorithm based on multi-feature is accomplished together with other feature-extracting actions [4, 5]. However, these methods directly replace the former Fourier transform through the wavelet transform to complete HRV signal filtering. This feature can contribute in improving the signal noise ratio; however, the analysis of cardiac autonomic nervous activity is lacking in many aspects. Consequently, a method that processes high sensitivity, low noise disturbance, and multi-scale information is needed. HRV signal is a complex signal that needs particular processing. Traditional methods fail in effective recognition. HRV signal is multi-scaled, chaotic, and non-stationary, and hence should be globally investigated to comprehend its essence. The wavelet entropy feature method offers a valid approach by combining multi-scale and non-linear analyses. The key to this method is the entropy value of the specific-scale wavelet coefficient. Inspired by the aforementioned factors, this method obtains the heart rate variation from the aberrant activity of the autonomic nervous system and further broadens the traditional wavelet entropy using the parameter *α*. This method is called *α*-entropy to signify the improved robustness and accuracy of its classification results and the controlled shortcomings using the time domain, frequency domain, and non-linear method. Moreover, the support vector machine (SVM) is used for classification; thus, PAF signals, signals distant from PAF, and normal sinus signal scan be accurately classified and identified.

## Methods

The primary hypothesis for AF is the exhumation mechanism, which states that other electricity conduction pathways, except the normal one, appear when PAF occurs. This phenomenon is objectively presented in the heart rate as a shorter interval with fast and irregular changes, and intermittent long intervals. Compared with the normal one, the HRV signal of PAF has increased complexity and chaos.

The diversity of HRV signals is mainly due to the different durations of the conduction of excitation regulation from different nerves, such as the antagonistic regulation of sympathetic and vagus nerves. Wavelet coefficients of HRV signal in specific scales can reflect the regulation on different time scales. In this paper, we use the *α* wavelet multi-scale entropy, which was developed from wavelet analysis and information entropy, and is used to quantitatively describe the information on different scales. The proposed PAF identification method is based on *α* wavelet multi-scale entropy and HRV analysis as follows:

To precisely obtain HRV signals, we should first eliminate the common noise, such as the 50 Hz power line interferences, electromyographic signals, and baseline wandering with band-pass filters in original ECG signals. Moreover, R waves can be located, and the distance of two adjacent R peaks can be calculated to procure the original HRV signals. The HRV sequences for subsequent analyses can be acquired after removing the possible artifacts and peculiar points.

**Fig. 1:**
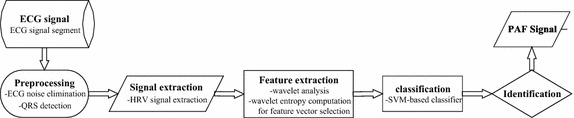
Algorithm process

The key step of PAF classification and identification is the extraction of the features, which are the traits of different electric cardiac activities of the HRV sequence. PAF often appears with arrhythmia; therefore, more tiny shakes will emerge in the HRV signals compared with the normal ones. Hence, PAF can be accurately identified by mapping this kind of shake to the corresponding scale-displacement space using multi-scale wavelet analysis and further characterizing the information quantity divergences from corresponding coefficient layer with multi-scale entropy. Algorithm process is shown in Fig. 1.

The wavelet multi-scale entropy of HRV signals can be computed as follows:

First, *m* scales discrete wavelet decomposition are created for HRV signals *f*(*t*). The discrete wavelet function *ϕ*_*j*,*k*_(*t*) is described as:

1 $$\phi_{jk} (t) = a_{0}^{{\frac{ - j}{2}}} \phi (a_{0}^{ - j} t - kb_{0} ), \quad a_{0} > 0,j \in N$$

where *a* = *a*_0_^−*j*^ is the scale factor and *b* = *kb*_0_ is the space factor. The wavelet transformation of *f*(*t*) can be:

2 $$W_{s} (a,t) = \frac{1}{{\sqrt {\left| a \right|} }}\int {f(t)\phi^{*} (t)dt ={\langle}{f(t).\phi_{at}{\rangle}}}$$

In multi-scale analysis, the wavelet function *ϕ*_*jk*_(*t*) can be obtained by processing $\varphi (t)$ through the combination of expansion, condensation, and shift. The quadrature wavelet function can be applied to decompose *f*(*t*) for the wavelet coefficient *D*_*jk*_:

3 $$f(t) = \sum\limits_{k = 1}^{n} {C_{jk} \varphi_{jk} (t) + \sum\limits_{j = 1}^{m} {\sum\limits_{k = 1}^{n} {D_{jk} \phi_{jk} (t)} } }$$

among which:

4 $$C_{jk} = {\langle}f(t),\varphi_{jk} (t){\rangle},\quad j,k \in z$$

5 $$D_{jk} = {\langle}f(t),\phi_{jk} (t){\rangle} ,\quad j,k \in z$$

where *j* represents the decomposition dimension and *k* represents the sampling time. The first item indicates the continuous approach of *f*(*t*) in scale *j* whose coefficient is called the discrete smoothing coefficient of *f*(*t*), whereas the second item is the detail of *f*(*t*) in scale *j*, and the corresponding coefficient *D*_*jk*_ is the wavelet coefficient, which is the intuitive estimate of regional power in different scales. The wavelet entropy in scale *j* can be defined as:

6 $$H_{j} = - \sum\limits_{k = 1}^{n} {p_{jk} \log p_{jk} }$$

where the coefficient *p*_*jk*_ can be calculated as:

7 $$p_{jk} = D_{jk} /\sum\limits_{k = 1}^{n} {D_{jk} }$$

Parameter *n* is the length of this scale wavelet coefficient. Equation (6) is the conventional definition of wavelet entropy. However, given that this method is sensible to noise, even a modicum of noise can yield great changes in the information quality, leading to incorrect estimation of HRV hallmarks. Accordingly, an improved method called *α*-order generalized entropy was proposed to compensate this disadvantage. The definition is depicted as:

8 $$H_{j} = \sum\limits_{k = 1}^{n} {\frac{{(p_{jk} )^{\alpha } - p_{jk} }}{\alpha - 1}}$$

Parameter *α* is a real number with values between 0–1 (not equal to 1). When *α* is set to 1, this generalized entropy reverts back to Shannon entropy. As a result, *α*-order generalized entropy can be seen as a generalization of Shannon entropy, and this definition is more applicable in describing the information quantity or level of chaos.

Hypothesis testing is used to pick up the scale wavelet entropy with the most statistic difference to build a feature vector for classification. The method of SVM is involved in completing the classification of PAF.

The support vector machine is a non-probabilistic binary linear classifier that belongs to the supervised learning model used for regression or classification. Assuming A-dimensional and S-sized dataset as:

9 $$\left\{ {\left( {x_{n} ,y_{n} } \right)\left| {x_{n} \in R^{A} ,y_{n} } \right. \in \left\{ { - 1, + 1} \right\}} \right\},n - 1, \ldots ,S$$

where n is the index of data. Each x_n_ is a A-dimensional vector, and y_n_ represents its corresponding class label. SVM will build a model through the following equations:

10 $$\mathop {\hbox{min} }\limits_{w,b} \frac{1}{2}\left\| w \right\|^{2}$$

11 $$s.t.y_{n} \left( {wx_{n} - b} \right) \ge 1$$

where **w** = [w] denotes the weights, and **b** = [b] denotes the biases. The distances between two hyper planes are 2/‖w‖; hence, Eq. (10) indicates that the distance between two hyper planes should be maximized. Equation (11) prevents the data falling to the margin as much as possible [6].

## Results

The data used in our study are from the MIT-BIH standard database and included 25 known samples of PAF segments, 25 segments distant from PAF, and 50 normal samples. Samples far away from PAF indicate an absence of PAF occurring in 45 min before and after sampling signals. The name marked with n as the initial is called a Normal signal (n08). The first letter p and last letter c suggest that the signal comes from an AF patient. If the middle digit is odd, then the signal is far away from PAF. Otherwise, the signal is a PAF signal. All signals are 5 min long and the sampling frequency is 128 Hz [7].

Before verifying the PAF recognition effect using our algorithm, the original ECG signals were required to remove the aforementioned interferences with a band-pass filter of 5–15 Hz; thus, the signals with their energy of QRS complexes were enhanced. Pan-Tompkin algorithm was used for locating the R waves. The HRV signals of 100 samples lasting 5 min were obtained.

Based on the high frequency of HRV signal (1 Hz) [8, 9], db8 was selected as the wavelet basis function, and eight scales of wavelet decomposition were created to ensure that each single-scale coefficient can map the corresponding excitability of cardiac electrical activity. Therefore, multi-scale wavelet entropy can be analyzed by combining meaningful physiological significance. The obtained eight-layer wavelet coefficients can be written as D1, D2,…, D7, and D8, respectively, where D1 is in the range of 0.5–1 Hz from the normalized frequency band and D2 ranges from 0.25 to 0.5 Hz. Similarly, the ranges of other coefficients decline in a binary manner according to the wavelet analysis theory. In light of the frequency-band partition rules of power spectrum, D2 and D3 wavelet coefficients reflected the high-frequency (HF) Section (0.15–0.4 Hz), which indicates the excitability of the vagus nerve; moreover, D4 and D5 wavelet coefficients represent the low-frequency(LF) Section (0.04–0.15 Hz), which mirrors the antagonism of the excitability of the sympathetic and vagus nerves; and the last three scales are the delegation of the very-low-frequency(VLF) Section (0–0.04 Hz) that is the indicator of the overall autonomic nervous irritability.

After the scale-entropy computation of each layer, the statistical *t*-test with 0.05 confidence was is used to select three layers with the most distinguishable statistical differences in identifying diverse PAF processes. These three chosen layers were considered as feature-layers and their respective wavelet entropy values were arranged as feature vectors of the later SVM-based fivefold cross validation to ensure the classification correction. The signal segments were randomly selected from the dataset and were not preselected to yield clear-cut PAF or normal sinus rhythm. The data from different individuals were used for training and not for evaluation. Repeated tests were completed for each single classification for 100 times to avouch the reliability and stability of the results.

Two kinds of experiments were conducted. One was the classification of PAF segments and segments distant from PAF, whereas the other was the identification of normal sinus heart rate signals and PAF signals. Three assessment indicators from clinical examination were used to evaluate the performance of our proposed method:

12 $$Correct\,Rate\,(\% ) = \frac{TP + TN}{TP + TN + FN + FP}*100$$

13 $$Sensitivity\,(\% ) = \frac{TP}{TP + FN}*100$$

14 $$Specificity\,(\% ) = \frac{TN}{TN + FP}*100$$

where TP, TN, FP, and FN stand for true positive, true negative, false positive, and false negative, respectively. The results were obtained after the generalized order and comparison with other HRV analysis methods.

Step 1.: Eliminating the common noise existing in original ECG signals;
Step 2.: Locating R waves and extracting HRV signals of samples;
Step 3.: Obtaining eight-layer wavelet coefficients written as D1, D2,…, D7, D8;
Step 4.: Computing the wavelet multi-scale entropy using Eq. 8
Step 5.: Selecting three layers using the 0.05 confidence *t* test
Step 6.: Conducting SVM-based fivefold cross validation 100 times to obtain the assessment indicators.

### Classification of PAF segments and segments distant from PAF

As discussed previously, *α*-order generalized entropy is the deduction of Shannon entropy. This entropy has a better universality for measuring the information quantity and chaos; hence, discussing the *α* value and ascertaining its optimal value were necessary.

The ergodic with 0.1 step size from 0.1 to 5.0 is used for *α* selection, during which each computed *α*-order wavelet entropy is applied to classify the PAF samples. For each value of *α*, tests will be repeated for 100 cycles; and the obtained correct rate, sensitivity, and specificity results of classification in the sense of mean and standard deviation are shown in the following figures (Fig. 1).

**Fig. 2:**
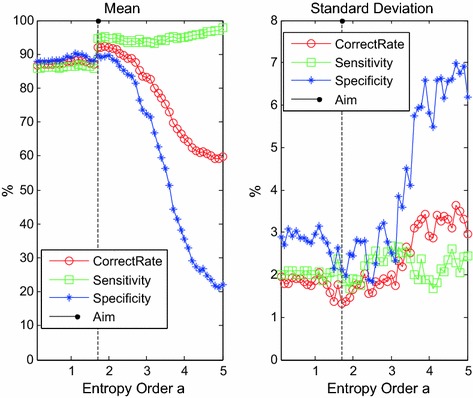
The mean and standard deviation of PAF classification with ergodic generalized entropy order *α*

From Fig. 2, the value of *α* remarkably influenced the correct rate of classification theoretically because different *α* values have unattached capacities to distinguish HRV signal and noise. When *α* is less than 1.5, all these three indicators are relatively stable and maintained high levels, thereby showing that the noise can be mistakenly identified as a meaningful signal in the case of small *α*. Although the overall classification correct rate is already stable around 86% and the error range is smaller than 2%, in terms of clinical application, high diagnosis accuracy is preferred because it lowers the medical cost and reduces the mental and physical burden of patients. When *α* is more than 3, the otherness of evaluation results is extant, which illustrates that a large *α* value will render the useful information of HRV signals to be determined as noise. Hence, the accuracy of classification declines markedly. When *α* is between 1.5 and 3, the wavelet entropy can effectively separate the valid information from HRV signals and noise. Here, *α* = 1.7 should be selected in this case because the peak value was approximately 1.7.

The figure below shows the wavelet entropy distribution of the layers of D2, D6, and D8 with *α* = 1.7, where S stands for PAF segments and T stands for segments that are distant from PAF.

**Fig. 3:**
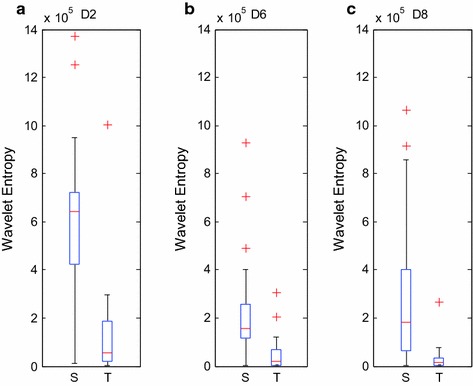
**a**–**c** show the classification box plot of HRV signals of the PAF segments and segments distant from PAF with D2, D6 and D8 wavelet entropy, respectively

Figure 3 shows that the values of the scale wavelet entropy of PAF segments are higher than the segments distant from PAF based on these three scales, which indicated that the wavelet entropy data distribution of the PAF segments and segments distant from PAF are remarkably distinguishable. This finding illustrates that the scale wavelet entropy used as the feature vector produces good distinction and can explain and represent the differentiation of the inner dynamic change pattern of PAF and non-PAF.

The entropy value of segments distant from PAF in Fig. 3a is lower than that of PAF segments because the irritability of the vagus nerve is enhanced in the PAF episode. Consequently, the conduction of electric excitability derived from cardiac atrionector is obstructed. Considering that this process might lead to the detainment of electric signals in the atrium, the activated “reentrant mechanism” can further induce the aberrant cardiac electric activity, which is expressed as an irregular tiny shake in HRV signals. As a consequence, the entire complexity of HRV signals will increase, thereby mapping the higher wavelet entropy value on the D6 or D8 scale in PAF episode.

### Classification of PAF-episode and sinus heart rate

**Fig. 4:**
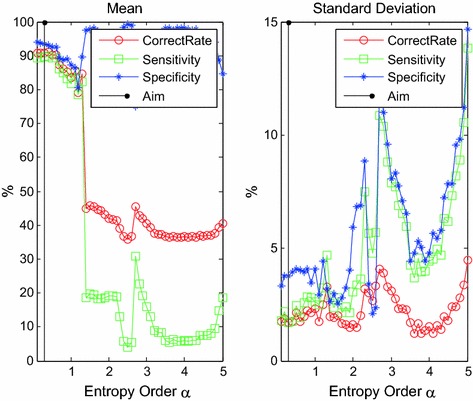
The mean and standard deviation of PAF classification with ergodic generalized entropy order *α*

Similarly, *α*-order wavelet entropy, computed through ergodic with 0.1 step size from 0.1 to 5.0, is used to classify the PAF episode and sinus heart rate for the perfect selection of *α*. For each value of *α*, the tests will be repeated circularly for 100 times, and the obtained correct rate, sensitivity, and specificity results of classification are shown in Fig. 4 as mean and standard deviation.

In Fig. 4, the correct rate of classification is remarkably affected by the value of *α*; however, some disparities were observed. When *α* is lower than 1.5, the correct rate, sensitivity, and specificity tend to decrease as *α* increases. The peak was found at approximately 0.3 because the normal sinus heart rate manifested differentiations in shake with the HRV signal of the PAF episode. A lower *α* value can separate the signal and noise effectively. By contrast, when *α* is higher than 1.5, the wanted information and noise should be undistinguished for wavelet entropy; moreover, declined classification accuracy and great fluctuation in deviation would be induced. Accordingly, *α* = 0.3 is the option for reckoning the generalized wavelet entropy and completing the classification of normal sinus heart rate and PAF episode.

The following figure is the box plots of D1, D2, and D8 layers of HRV signals of PAF episode and normal sinus heart rate when *α* = 0.3.

**Fig. 5:**
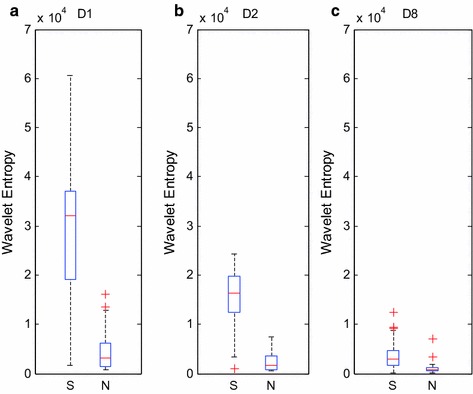
**a**–**c** show the classification box plot of HRV signals of PAF-episode and normal ones with D1, D2 and D8 wavelet entropy, respectively, where S stands for PAF segments, while T stands for normal ones

Figure 5 shows that the wavelet entropy value of PAF-episode is higher than the normal sinus heart rate signals; hence, the multi-scale wavelet entropy can sensitively capture the cardiac activity differentiation result from the AF episode, and the differentiation can be seen as a feature to distinguish the signals of the sinus heart rate and PAF. The option of wavelet coefficients of D1, D2, and D8-scale are different from the former experiment due to the divergence in the HRV signal of the sinus heart rate and PAF and the expression of arrhythmic AF episode in the HF wavelet coefficient. This finding is the main reason for selecting D1, whereas the choices of D2 and D8 scale were the same in the former experiment.

## Discussion

### Classification of PAF segments and segments distant from PAF

Four other common HRV signal analysis algorithms are presented and compared to display the superiority and characteristic of multi-scale wavelet entropy.

Time-domain feature; with major four parameters as mean, SDNN, RMSSD, and pNN50;

Frequency-domain feature; with major four parameters as pVLF, pLF, pHF, and ratio;

Sample-entropy feature; since this method need parameter value selection, m = 2, r, which is the half of sample standard deviation.

The wavelet power feature mainly involves eight parameters (the same db8-scale-based wavelet analysis, the obtained wavelet coefficient energy of each scale are chosen as parameters).

The classification results of these four methods with the proposed methods of PAF segment and segments distant from PAF are shown as follows:

The proposed multi-scale-wavelet entropy-based method is dominant in correct rate, specificity, and sensitivity for classification compared with others, as shown in Table 1, thereby manifesting the great performance of this method in obtaining the dynamic change process and fundamental characteristics of AF episode electric activity. As mentioned, a PAF episode tends to display irregular tiny shakes compared with the segments distant from PAF. The differentiation in this shake pattern can adequately map HRV signal characteristics to the corresponding scale-displacement space, which can be expressed as the divergence of distribution information of the corresponding coefficient scale. Based on this feature, the PAF identification is valid.

**Table 1:** Classification results of *α* = 0.3 and methods based on wavelet entropy feature, time-domain feature, frequency-domain feature, and wavelet-energy feature

	Correct rate (%)	Sensitivity (%)	Specificity (%)
Time	82.20 ± 2.04	77.68 ± 1.98	86.72 ± 3.50
Frequency	57.96 ± 4.93	48.84 ± 6.63	67.08 ± 7.75
SampEn	64.32 ± 1.96	76.36 ± 3.90	52.28 ± 1.03
Wavelet Energy	87.50 ± 2.10	93.68 ± 2.68	81.32 ± 3.12
Wavelet Entropy	*91.98* ± *1.32*	*94.64* ± *1.90*	*89.32* ± *1.89*

Italic values indicate the highest classification correct rate, specificity or sensitivity among different methods

Table 1 shows that although the time-domain feature method has good overall performance; moreover, the lack of time temporal association information analysis might lessen its classification ability compared with the wavelet-entropy feature. The time-domain feature method is easily affected by the noise from samples and usually lacks stability when the fluctuation of standard deviation exceeds 3%. The frequency-domain feature method is affected by the hypothesis that HRV signals are approximately stable, which results in the bad manifestation in all the three indicators with low stability. Theoretically, the sample-entropy feature based on the non-linear theory is the best method; however, this technique is highly sensitive to noise and hence its classification capability is weakened, even if the classification standard fluctuation is small. However, the wavelet analysis overcoming the defects of traditional frequency-domain feature method has marked improvements in correct rate, sensitivity, and specificity. The multi-scale wavelet entropy method combining the characteristics of wavelet analysis and non-linear feature analysis can map the shake trait of the HRV signal to the energy information distribution of the multi-scale space. Therefore, using the wavelet-entropy-based method to classify PAF segments and segments distant from PAF has some advancement and certain application prospects.

### Classification of PAF-episode and sinus heart rate

Homoplastically, compared with the other four methods, the results from this proposed algorithm coupled with SVM-based classifier are as follows:

As shown in Table 2, this proposed wavelet multi-scale *α* entropy-based method has conspicuous dominance in the correct rate, specificity, and sensitivity for sorting the signals of PAF episode and normal sinus heart rate compared with others. The results reveal the great performance of our method in attaining the dynamic change process and the basis characteristics of AF episode electric activity. Different from the result of former experiment, the time-domain feature method has a 87% correct rate and 91% sensitivity, mainly because the HRV signal of PAF episode displays fiercer shakes than normal sinus heart rate ones, thereby increasing the irrelevance among different spots. As a result, the defect of lacking the temporal sequence association information analysis in the time-domain feature method is controlled; hence, the correct rate and sensitivity are the prime. However, the specificity cannot be increased. After selecting the appropriate *α*, the proposed algorithm can achieve high recognition correct rate and specificity for PAF segment and normal sinus segment, as well as high sensitivity.

**Table 2:** Classification results of *α* = 0.3 and methods based on wavelet entropy feature, time-domain feature, frequency-domain feature, and wavelet-energy feature

	Correct rate (%)	Sensitivity (%)	Specificity (%)
Time	87.40 ± 2.06	*91.42* ± *2.55*	79.36 ± 3.10
Frequency	69.51 ± 3.63	68.24 ± 4.88	72.04 ± 4.97
SampEn	66.88 ± 1.34	74.62 ± 1.77	51.40 ± 1.54
Wavelet Energy	40.40 ± 1.84	17.04 ± 3.32	87.12 ± 5.90
Wavelet Entropy	*91.12* *±* *1.86*	89.86 ± 2.04	*93.64* ± *3.60*

Italic values indicate the highest classification correct rate, specificity or sensitivity among different methods

These two experiments suggested that multi-scale wavelet entropy has great sensitivity for changing cardiac electric activity from different PAF processes. By mapping the HRV signals to different scale spaces, the radical features of its change in shaking can be seized, and the decent identification rate can be secured by selecting the proper *α* to describe the features.

E Sabeti et al. used intrinsic mode functions (IMFs) and physiological features, such as the number of premature beats (PBs), to predict the onset of PAF [10] and thus achieved the correct rate of 88%. M Udd in Ahmed et al. used the multivariate multi-scale entropy to analyze the biological recordings [11].The method in this article is more accurate and has lower computational complexity than those in the above studies.

## Conclusions

This paper analyzed the wavelet multi-scale *α*-entropy that reflects the regulation of sympathetic and vagus nerves in the HRV signal, which fully embodies the information discrepancy in various HRV signals. Most of the physiological differences between PAF segments, segments distant from PAF segments, and normal sinus heart rate signals are sufficiently reflected by the different appropriate scales of wavelet entropy, which are necessarily computed and selected as the extracted features. Research results revealed that generalization wavelet entropy, as a method to analyze HRV signals, is competent for acquiring the differentiation of an inner dynamic change pattern of PAF and non-PAF and has an application value in clinical diagnosis, treatment, and monitoring AF.

## Abbreviations

PAF: paroxysmal atrial fibrillation
HRV: heart rate variability
ECG: electrocardiograph
SVM: support vector machine
AF: atrial fibrillation
WT: wavelet transform
EMD: empirical mode decomposition
HF: high-frequency
LF: low-frequency
VLF: very-low-frequency
